# (*Z*)-*N*-[5-Bromo-2-(4-methyl­anilino)-3*H*-indol-3-yl­idene]-4-methyl­aniline oxide

**DOI:** 10.1107/S1600536811000833

**Published:** 2011-01-12

**Authors:** Davood Asgri, Mohammad Ghanbari, Morteza Mehrdad, Khosrow Jadidi, Hamid Reza Khavasi

**Affiliations:** a Departement of Chemistry, Shahid Beheshti University, G. C., Evin, Tehran 1983963113, Iran; b Department of Environmental Pollution, Environmental Sciences Research Institute, Shahid Beheshti University, G.C., Evin, Tehran 1983963113, Iran

## Abstract

The crystal structure of the title compound, C_22_H_18_BrN_3_O, is stabilized by π–π contacts [centroid–centroid distance = 3.476 (2) Å] between five-membered rings as well as inter­molecular C—H⋯O and C—H⋯N hydrogen bonds. An intra­molecular N—H⋯O hydrogen bond occurs. The benzene rings make a dihedral angle of 59.89 (8)°. The dihedral angles between the fused ring systemand the two benzene rings are 3.46 (7) and 61.97 (7)°.

## Related literature

For background to this study and a related structure, see: Mehrdad *et al.* (2011 ▶). For the Baeyer–Villiger oxidation of 1-alkyl-3-arylimino-2-indolinones, see: Jadidi *et al.* (2008 ▶); Azizian *et al.* (2010 ▶).
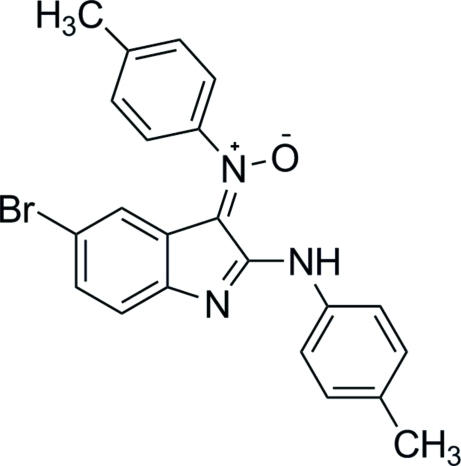


## Experimental

### 

#### Crystal data

C_22_H_18_BrN_3_O
*M* *_r_* = 420.29
Triclinic, 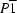

*a* = 7.8676 (4) Å
*b* = 8.9748 (4) Å
*c* = 14.1353 (7) Å
α = 83.090 (4)°
β = 74.363 (4)°
γ = 69.776 (4)°
*V* = 901.49 (8) Å^3^
*Z* = 2
Mo *K*α radiation
μ = 2.30 mm^−1^
*T* = 298 K
0.3 × 0.16 × 0.11 mm

#### Data collection

Stoe IPDS IIT diffractometer
Absorption correction: numerical [shape of crystal determined optically (*X-RED* and *X-SHAPE*; Stoe & Cie, 2005 ▶) *T*_min_ = 0.681, *T*_max_ = 0.805
9841 measured reflections
4839 independent reflections
4382 reflections with *I* > 2σ(*I*)
*R*_int_ = 0.049

#### Refinement

*R*[*F* ^2^ > 2σ(*F* ^2^)] = 0.045
*wR*(*F* ^2^) = 0.119
*S* = 1.10
4839 reflections
244 parameters
H-atom parameters constrained
Δρ_max_ = 0.91 e Å^−3^
Δρ_min_ = −1.10 e Å^−3^

### 

Data collection: *X-AREA* (Stoe & Cie, 2005 ▶); cell refinement: *X-AREA*; data reduction: *X-AREA*; program(s) used to solve structure: *SHELXS97* (Sheldrick, 2008 ▶); program(s) used to refine structure: *SHELXL97* (Sheldrick, 2008 ▶); molecular graphics: *ORTEP-3 for Windows* (Farrugia, 1997 ▶); software used to prepare material for publication: *WinGX* (Farrugia, 1999 ▶).

## Supplementary Material

Crystal structure: contains datablocks global, I. DOI: 10.1107/S1600536811000833/bt5458sup1.cif

Structure factors: contains datablocks I. DOI: 10.1107/S1600536811000833/bt5458Isup2.hkl

Additional supplementary materials:  crystallographic information; 3D view; checkCIF report

## Figures and Tables

**Table 1:** Hydrogen-bond geometry (Å, °)

*D*—H⋯*A*	*D*—H	H⋯*A*	*D*⋯*A*	*D*—H⋯*A*
N1—H1*B*⋯O1	0.86	1.99	2.677 (3)	137
C16—H16⋯N3^i^	0.93	2.45	3.369 (3)	169
C20—H20⋯O1^ii^	0.93	2.62	3.434 (3)	146

Symmetry codes: (i) 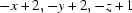; (ii) 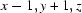.

**Fig. 1.**
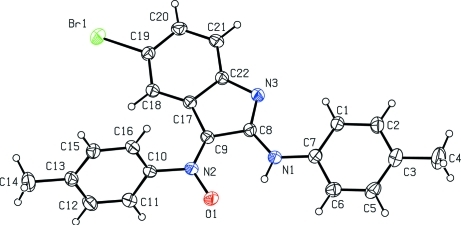
The molecular structure of the title molecule, with the atom-numbering scheme. Displacement ellipsoids are drawn at the 30% probability level.

## supplementary crystallographic information

### Comment

The background to this study is set out in the preceding paper (Mehrdad *et al.*, 2011). In this paper, we report the structure of *N*-aryl-*N*-(2-arylamino-3*H*-indol-3-ylidene) amine N-oxide. The molecular structure of the title compound is shown in Fig. 1.

In the molecules of the title compound, bond distances and angles are within normal ranges.

The dihedral angles between the rings are A (C1—C7), B (C10—C16) and  C (C17—C22/N3/C8/C9) are A/B = 59.89 (8)°, A/C = 3.46 (7)° and B/C = 61.97 (7)°. A packing diagram is shown in Fig. 2. Intermolecular C—H···N contacts and π–π interactions between 5-membered rings [centroid···centroid distance = 3.476 (2) Å; symmetry operator 2-x,2-y,1-z] stabilize the crystal structure.

### Experimental

The preparation of the title compound has been reported previously, except that the temperature was room temperature in place of -20°C (Mehrdad *et al.*, 2010). At -20°C (2Z)-N-(4-Methoxyphenyl)-2-(4-methoxyphenylimino)-2H-1,4-benzoxazin-3-amine was obtained, whereas at  room temperature (*Z*)—*N*-(5-bromo-2-(*p*-tolylamino)-3*H*-indol-3- ylidene)-4-methylaniline oxide was obtained. It was isolated as a violet solid in 72% yield: m.p. = 183–184 C.

### Refinement

All H atoms were positioned geometrically, with N—H=0.86 Å, C—H=0.96Å and C—H=0.93Åfor N—H, aromatics and methyl hydrogen atoms respectively and constrained to ride on their parent atoms, with *U*_iso_(H)=1.2*U*_eq_.

### Crystal data

**Table:**

C_22_H_18_BrN_3_O	*Z* = 2
*M**_r_* = 420.29	*F*(000) = 428
Triclinic, *P*1	*D*_x_ = 1.548 Mg m^−^^3^
Hall symbol: -P 1	Mo *K*α radiation, λ = 0.71073 Å
*a* = 7.8676 (4) Å	Cell parameters from 9841 reflections
*b* = 8.9748 (4) Å	θ = 2.4–29.1°
*c* = 14.1353 (7) Å	µ = 2.30 mm^−^^1^
α = 83.090 (4)°	*T* = 298 K
β = 74.363 (4)°	Prism, red
γ = 69.776 (4)°	0.3 × 0.16 × 0.11 mm
*V* = 901.49 (8) Å^3^	

### Data collection

**Table:**

Stoe IPDS IIT diffractometer	4839 independent reflections
graphite	4382 reflections with *I* > 2σ(*I*)
Detector resolution: 0.15 mm pixels mm^-1^	*R*_int_ = 0.049
rotation method scans	θ_max_ = 29.1°, θ_min_ = 2.4°
Absorption correction: numerical [shape of crystal determined optically (*X-RED* and *X-SHAPE*; Stoe & Cie, 2005)	*h* = −10→10
*T*_min_ = 0.681, *T*_max_ = 0.805	*k* = −12→12
9841 measured reflections	*l* = −19→14

### Refinement

**Table:**

Refinement on *F*^2^	Primary atom site location: structure-invariant direct methods
Least-squares matrix: full	Secondary atom site location: difference Fourier map
*R*[*F*^2^ > 2σ(*F*^2^)] = 0.045	Hydrogen site location: inferred from neighbouring sites
*wR*(*F*^2^) = 0.119	H-atom parameters constrained
*S* = 1.10	*w* = 1/[σ^2^(*F*_o_^2^) + (0.0603*P*)^2^ + 1.2665*P*] where *P* = (*F*_o_^2^ + 2*F*_c_^2^)/3
4839 reflections	(Δ/σ)_max_ = 0.008
244 parameters	Δρ_max_ = 0.91 e Å^−^^3^
0 restraints	Δρ_min_ = −1.10 e Å^−^^3^

### Special details

**Table:**

Geometry. All e.s.d.'s (except the e.s.d. in the dihedral angle between two l.s. planes) are estimated using the full covariance matrix. The cell e.s.d.'s are taken into account individually in the estimation of e.s.d.'s in distances, angles and torsion angles; correlations between e.s.d.'s in cell parameters are only used when they are defined by crystal symmetry. An approximate (isotropic) treatment of cell e.s.d.'s is used for estimating e.s.d.'s involving l.s. planes.
Refinement. Refinement of *F*^2^ against ALL reflections. The weighted *R*-factor *wR* and goodness of fit *S* are based on *F*^2^, conventional *R*-factors *R* are based on *F*, with *F* set to zero for negative *F*^2^. The threshold expression of *F*^2^ > σ(*F*^2^) is used only for calculating *R*-factors(gt) *etc*. and is not relevant to the choice of reflections for refinement. *R*-factors based on *F*^2^ are statistically about twice as large as those based on *F*, and *R*- factors based on ALL data will be even larger.

### Fractional atomic coordinates and isotropic or equivalent isotropic displacement parameters (Å^2^)

**Table:**

	*x*	*y*	*z*	*U*_iso_*/*U*_eq_	
C1	1.0280 (4)	0.6363 (3)	0.3135 (2)	0.0203 (5)	
H1	0.9265	0.7273	0.3328	0.024*	
C2	1.0624 (4)	0.5714 (3)	0.2226 (2)	0.0219 (5)	
H2	0.9835	0.6211	0.1814	0.026*	
C3	1.2118 (4)	0.4339 (3)	0.1920 (2)	0.0208 (5)	
C4	1.2539 (4)	0.3684 (4)	0.0918 (2)	0.0288 (6)	
H4A	1.2522	0.2611	0.099	0.035*	
H4B	1.3751	0.3699	0.0548	0.035*	
H4C	1.1613	0.4324	0.0581	0.035*	
C5	1.3260 (4)	0.3607 (3)	0.2564 (2)	0.0219 (5)	
H5	1.4245	0.2673	0.2384	0.026*	
C6	1.2958 (4)	0.4239 (3)	0.3460 (2)	0.0199 (5)	
H6	1.3745	0.3736	0.3873	0.024*	
C7	1.1468 (3)	0.5638 (3)	0.37508 (19)	0.0173 (4)	
C8	0.9991 (3)	0.7513 (3)	0.51299 (19)	0.0162 (4)	
C9	1.0160 (3)	0.7961 (3)	0.60744 (19)	0.0167 (4)	
C10	1.1776 (3)	0.7865 (3)	0.73329 (19)	0.0169 (4)	
C11	1.1803 (4)	0.6950 (3)	0.8196 (2)	0.0196 (5)	
H11	1.1661	0.5956	0.8241	0.023*	
C12	1.2048 (4)	0.7557 (3)	0.8991 (2)	0.0206 (5)	
H12	1.2046	0.6967	0.9579	0.025*	
C13	1.2295 (4)	0.9033 (3)	0.89224 (19)	0.0195 (5)	
C14	1.2576 (4)	0.9686 (4)	0.9787 (2)	0.0268 (6)	
H14A	1.1625	1.0694	0.9953	0.032*	
H14B	1.3783	0.9819	0.9616	0.032*	
H14C	1.2499	0.8959	1.0341	0.032*	
C15	1.2301 (4)	0.9902 (3)	0.8033 (2)	0.0194 (5)	
H15	1.2478	1.0884	0.7977	0.023*	
C16	1.2046 (4)	0.9327 (3)	0.7233 (2)	0.0192 (5)	
H16	1.2056	0.991	0.6642	0.023*	
C17	0.8539 (3)	0.9366 (3)	0.63666 (19)	0.0159 (4)	
C18	0.7762 (3)	1.0326 (3)	0.71857 (18)	0.0174 (5)	
H18	0.8311	1.0128	0.7714	0.021*	
C19	0.6130 (3)	1.1593 (3)	0.71750 (19)	0.0171 (4)	
C20	0.5291 (4)	1.1944 (3)	0.6390 (2)	0.0190 (5)	
H20	0.4235	1.2832	0.64	0.023*	
C21	0.6048 (4)	1.0953 (3)	0.55863 (19)	0.0189 (5)	
H21	0.5489	1.1161	0.5061	0.023*	
C22	0.7650 (3)	0.9650 (3)	0.55820 (18)	0.0166 (4)	
N1	1.1289 (3)	0.6222 (3)	0.46644 (16)	0.0177 (4)	
H1B	1.2127	0.568	0.497	0.021*	
N2	1.1553 (3)	0.7223 (2)	0.64904 (17)	0.0176 (4)	
N3	0.8540 (3)	0.8514 (2)	0.48464 (16)	0.0166 (4)	
O1	1.2865 (3)	0.5915 (2)	0.61671 (15)	0.0233 (4)	
Br1	0.49974 (4)	1.28997 (3)	0.82884 (2)	0.02254 (10)	

### Atomic displacement parameters (Å^2^)

**Table:**

	*U*^11^	*U*^22^	*U*^33^	*U*^12^	*U*^13^	*U*^23^
C1	0.0190 (11)	0.0149 (11)	0.0265 (13)	−0.0007 (9)	−0.0084 (10)	−0.0072 (9)
C2	0.0230 (12)	0.0170 (11)	0.0264 (13)	−0.0033 (10)	−0.0090 (10)	−0.0068 (10)
C3	0.0185 (11)	0.0188 (11)	0.0251 (12)	−0.0055 (9)	−0.0030 (10)	−0.0084 (10)
C4	0.0300 (14)	0.0300 (14)	0.0257 (13)	−0.0073 (12)	−0.0032 (11)	−0.0138 (11)
C5	0.0164 (11)	0.0164 (11)	0.0292 (13)	−0.0019 (9)	−0.0014 (10)	−0.0072 (10)
C6	0.0166 (11)	0.0148 (11)	0.0255 (12)	−0.0013 (9)	−0.0046 (9)	−0.0033 (9)
C7	0.0163 (10)	0.0136 (10)	0.0218 (11)	−0.0038 (9)	−0.0036 (9)	−0.0052 (9)
C8	0.0172 (11)	0.0115 (10)	0.0200 (11)	−0.0044 (8)	−0.0041 (9)	−0.0034 (8)
C9	0.0162 (11)	0.0125 (10)	0.0211 (11)	−0.0024 (8)	−0.0063 (9)	−0.0019 (8)
C10	0.0149 (10)	0.0142 (10)	0.0208 (11)	−0.0005 (8)	−0.0075 (9)	−0.0037 (8)
C11	0.0207 (11)	0.0134 (10)	0.0250 (12)	−0.0042 (9)	−0.0083 (10)	−0.0003 (9)
C12	0.0210 (12)	0.0208 (12)	0.0193 (11)	−0.0039 (10)	−0.0078 (9)	−0.0002 (9)
C13	0.0158 (11)	0.0198 (11)	0.0205 (12)	−0.0009 (9)	−0.0041 (9)	−0.0077 (9)
C14	0.0275 (14)	0.0301 (14)	0.0241 (13)	−0.0058 (11)	−0.0096 (11)	−0.0094 (11)
C15	0.0189 (11)	0.0134 (10)	0.0252 (12)	−0.0028 (9)	−0.0064 (10)	−0.0033 (9)
C16	0.0205 (11)	0.0141 (10)	0.0215 (12)	−0.0026 (9)	−0.0065 (9)	−0.0016 (9)
C17	0.0148 (10)	0.0127 (10)	0.0205 (11)	−0.0034 (8)	−0.0054 (9)	−0.0026 (8)
C18	0.0182 (11)	0.0156 (11)	0.0178 (11)	−0.0035 (9)	−0.0053 (9)	−0.0033 (8)
C19	0.0154 (10)	0.0131 (10)	0.0207 (11)	−0.0022 (8)	−0.0013 (9)	−0.0069 (8)
C20	0.0175 (11)	0.0146 (10)	0.0241 (12)	−0.0022 (9)	−0.0071 (9)	−0.0020 (9)
C21	0.0191 (11)	0.0160 (11)	0.0215 (12)	−0.0038 (9)	−0.0075 (9)	−0.0008 (9)
C22	0.0178 (11)	0.0146 (10)	0.0184 (11)	−0.0058 (9)	−0.0051 (9)	−0.0016 (8)
N1	0.0170 (9)	0.0148 (9)	0.0209 (10)	−0.0016 (8)	−0.0069 (8)	−0.0040 (8)
N2	0.0165 (9)	0.0119 (9)	0.0234 (10)	−0.0010 (7)	−0.0070 (8)	−0.0027 (8)
N3	0.0175 (9)	0.0137 (9)	0.0180 (9)	−0.0031 (8)	−0.0048 (8)	−0.0035 (7)
O1	0.0207 (9)	0.0149 (8)	0.0308 (10)	0.0043 (7)	−0.0107 (8)	−0.0089 (7)
Br1	0.02190 (14)	0.01708 (13)	0.02465 (15)	−0.00021 (9)	−0.00401 (10)	−0.00834 (9)

### Geometric parameters (Å, °)

**Table:**

C1—C7	1.391 (4)	C11—H11	0.93
C1—C2	1.396 (4)	C12—C13	1.393 (4)
C1—H1	0.93	C12—H12	0.93
C2—C3	1.394 (4)	C13—C15	1.397 (4)
C2—H2	0.93	C13—C14	1.515 (4)
C3—C5	1.398 (4)	C14—H14A	0.96
C3—C4	1.507 (4)	C14—H14B	0.96
C4—H4A	0.96	C14—H14C	0.96
C4—H4B	0.96	C15—C16	1.385 (4)
C4—H4C	0.96	C15—H15	0.93
C5—C6	1.378 (4)	C16—H16	0.93
C5—H5	0.93	C17—C18	1.397 (3)
C6—C7	1.403 (3)	C17—C22	1.418 (3)
C6—H6	0.93	C18—C19	1.393 (3)
C7—N1	1.406 (3)	C18—H18	0.93
C8—N3	1.312 (3)	C19—C20	1.391 (4)
C8—N1	1.349 (3)	C19—Br1	1.901 (2)
C8—C9	1.491 (3)	C20—C21	1.395 (4)
C9—N2	1.318 (3)	C20—H20	0.93
C9—C17	1.455 (3)	C21—C22	1.391 (3)
C10—C16	1.385 (4)	C21—H21	0.93
C10—C11	1.387 (4)	C22—N3	1.407 (3)
C10—N2	1.459 (3)	N1—H1B	0.86
C11—C12	1.390 (4)	N2—O1	1.300 (3)
			
C7—C1—C2	119.5 (2)	C12—C13—C14	121.1 (3)
C7—C1—H1	120.2	C15—C13—C14	120.3 (2)
C2—C1—H1	120.2	C13—C14—H14A	109.5
C3—C2—C1	121.7 (3)	C13—C14—H14B	109.5
C3—C2—H2	119.1	H14A—C14—H14B	109.5
C1—C2—H2	119.1	C13—C14—H14C	109.5
C2—C3—C5	117.7 (2)	H14A—C14—H14C	109.5
C2—C3—C4	121.6 (3)	H14B—C14—H14C	109.5
C5—C3—C4	120.7 (2)	C16—C15—C13	121.2 (2)
C3—C4—H4A	109.5	C16—C15—H15	119.4
C3—C4—H4B	109.5	C13—C15—H15	119.4
H4A—C4—H4B	109.5	C15—C16—C10	118.5 (2)
C3—C4—H4C	109.5	C15—C16—H16	120.8
H4A—C4—H4C	109.5	C10—C16—H16	120.8
H4B—C4—H4C	109.5	C18—C17—C22	120.8 (2)
C6—C5—C3	121.5 (2)	C18—C17—C9	135.5 (2)
C6—C5—H5	119.2	C22—C17—C9	103.6 (2)
C3—C5—H5	119.2	C19—C18—C17	117.0 (2)
C5—C6—C7	120.1 (2)	C19—C18—H18	121.5
C5—C6—H6	119.9	C17—C18—H18	121.5
C7—C6—H6	119.9	C20—C19—C18	123.1 (2)
C1—C7—C6	119.4 (2)	C20—C19—Br1	118.58 (18)
C1—C7—N1	124.2 (2)	C18—C19—Br1	118.31 (19)
C6—C7—N1	116.4 (2)	C19—C20—C21	119.5 (2)
N3—C8—N1	128.1 (2)	C19—C20—H20	120.3
N3—C8—C9	112.3 (2)	C21—C20—H20	120.3
N1—C8—C9	119.6 (2)	C22—C21—C20	119.1 (2)
N2—C9—C17	130.5 (2)	C22—C21—H21	120.5
N2—C9—C8	124.9 (2)	C20—C21—H21	120.5
C17—C9—C8	104.6 (2)	C21—C22—N3	125.9 (2)
C16—C10—C11	122.1 (2)	C21—C22—C17	120.4 (2)
C16—C10—N2	119.2 (2)	N3—C22—C17	113.7 (2)
C11—C10—N2	118.6 (2)	C8—N1—C7	129.2 (2)
C10—C11—C12	118.4 (2)	C8—N1—H1B	115.4
C10—C11—H11	120.8	C7—N1—H1B	115.4
C12—C11—H11	120.8	O1—N2—C9	123.3 (2)
C11—C12—C13	121.2 (2)	O1—N2—C10	114.8 (2)
C11—C12—H12	119.4	C9—N2—C10	121.8 (2)
C13—C12—H12	119.4	C8—N3—C22	105.6 (2)
C12—C13—C15	118.7 (2)		
			
C7—C1—C2—C3	0.9 (4)	C9—C17—C18—C19	−179.4 (3)
C1—C2—C3—C5	1.0 (4)	C17—C18—C19—C20	−1.3 (4)
C1—C2—C3—C4	−177.1 (3)	C17—C18—C19—Br1	178.59 (18)
C2—C3—C5—C6	−1.7 (4)	C18—C19—C20—C21	3.1 (4)
C4—C3—C5—C6	176.3 (3)	Br1—C19—C20—C21	−176.81 (19)
C3—C5—C6—C7	0.7 (4)	C19—C20—C21—C22	−1.3 (4)
C2—C1—C7—C6	−1.9 (4)	C20—C21—C22—N3	177.6 (2)
C2—C1—C7—N1	177.7 (3)	C20—C21—C22—C17	−2.2 (4)
C5—C6—C7—C1	1.2 (4)	C18—C17—C22—C21	4.0 (4)
C5—C6—C7—N1	−178.5 (2)	C9—C17—C22—C21	−178.0 (2)
N3—C8—C9—N2	−175.3 (2)	C18—C17—C22—N3	−175.8 (2)
N1—C8—C9—N2	3.0 (4)	C9—C17—C22—N3	2.2 (3)
N3—C8—C9—C17	2.5 (3)	N3—C8—N1—C7	2.1 (4)
N1—C8—C9—C17	−179.2 (2)	C9—C8—N1—C7	−175.9 (2)
C16—C10—C11—C12	2.2 (4)	C1—C7—N1—C8	1.4 (4)
N2—C10—C11—C12	179.0 (2)	C6—C7—N1—C8	−178.9 (3)
C10—C11—C12—C13	−1.2 (4)	C17—C9—N2—O1	176.1 (2)
C11—C12—C13—C15	−0.2 (4)	C8—C9—N2—O1	−6.7 (4)
C11—C12—C13—C14	−179.5 (2)	C17—C9—N2—C10	−6.7 (4)
C12—C13—C15—C16	0.7 (4)	C8—C9—N2—C10	170.5 (2)
C14—C13—C15—C16	180.0 (2)	C16—C10—N2—O1	115.9 (3)
C13—C15—C16—C10	0.3 (4)	C11—C10—N2—O1	−60.9 (3)
C11—C10—C16—C15	−1.7 (4)	C16—C10—N2—C9	−61.5 (3)
N2—C10—C16—C15	−178.5 (2)	C11—C10—N2—C9	121.7 (3)
N2—C9—C17—C18	−7.4 (5)	N1—C8—N3—C22	−179.3 (3)
C8—C9—C17—C18	175.0 (3)	C9—C8—N3—C22	−1.2 (3)
N2—C9—C17—C22	175.1 (3)	C21—C22—N3—C8	179.5 (3)
C8—C9—C17—C22	−2.6 (3)	C17—C22—N3—C8	−0.6 (3)
C22—C17—C18—C19	−2.2 (4)		

### Hydrogen-bond geometry (Å, °)

**Table:**

*D*—H···*A*	*D*—H	H···*A*	*D*···*A*	*D*—H···*A*
N1—H1B···O1	0.86	1.99	2.677 (3)	137
C16—H16···N3^i^	0.93	2.45	3.369 (3)	169
C20—H20···O1^ii^	0.93	2.62	3.434 (3)	146

Symmetry codes: (i) −*x*+2, −*y*+2, −*z*+1; (ii) *x*−1, *y*+1, *z*.
